# Using multivariate nonlinear mixed-effects model to investigate factors influencing symptom improvement after high tibial osteotomy in combination with bone marrow concentrate injection for medial compartment knee osteoarthritis: a prospective, open-label study

**DOI:** 10.1186/s12891-023-06314-z

**Published:** 2023-03-20

**Authors:** Hsiao-Yi Cheng, Chun-Wei Liang, Chen-Lun Chu, Hao-Wei Hsu, Sheng-Mou Hou, Kao-Shang Shih

**Affiliations:** 1 Department of Primary Care Medicine, Shin Kong Wu Ho-Su Memorial Hospital, Taipei, Taiwan; 2 School of Medicine, College of Medicine, Taipei Medical University, Taipei, Taiwan; 3 Department of Orthopaedic Surgery, Shin Kong Wu Ho-Su Memorial Hospital, Taipei, Taiwan; 4 School of Medicine, Fu-Jen Catholic University, New Taipei City, Taiwan

**Keywords:** High tibial osteotomy, Bone marrow concentrate, Varus knee, Knee osteoarthritis

## Abstract

**Purpose:**

To investigate the effects of various demographic, structural, radiographic, and clinical factors on the prognosis of patients with medial compartmental knee osteoarthritis with varus deformity undergoing medial opening wedge high tibial osteotomy (HTO) in combination with bone marrow concentrate (BMC) injection.

**Methods:**

In this prospective study, 20 patients underwent medial opening wedge HTO in combination with BMC injection with 12 months of follow-up. The structural and radiographic outcomes were evaluated by femorotibial angle and posterior tibial slope angle. The clinical outcomes were evaluated by visual analogue scale (VAS), Western Ontario and McMaster Universities Arthritis Index (WOMAC), and The Knee injury and Osteoarthritis Outcome Score (KOOS). Multivariate nonlinear mixed-effects models with asymptotic regressions were used to model the trajectory of symptom improvement.

**Results:**

Medial opening wedge HTO in combination with BMC corrected the malalignment of the knee and led to significant symptom relief. The improvement of clinical symptoms reached a plateau 6 months after the surgery. Greater symptom severity at baseline and lower Kellgren-Lawrance (KL) grades were correlated with better post-operative clinical outcomes. Body-Mass-Index (BMI), femorotibial angle, age, and sex may also play a role in influencing the extent of symptom relief.

**Conclusion:**

Symptom severity at baseline is important for prognosis prediction. In clinical practice, we suggest that the evaluation of clinical features and functional status of the patients be more emphasised.

**Supplementary Information:**

The online version contains supplementary material available at 10.1186/s12891-023-06314-z.

## Introduction

Knee osteoarthritis is one of the leading causes of knee pain and dysfunction [1, 2]. Because of the ageing population and the prevalence of obesity, the increase in global incidence rate of knee osteoarthritis has led to severe physical, psychological, and economic burden on patients’ families and the whole society [3–6]. According to the current European Society for Clinical and Economic Aspects of Osteoporosis, Osteoarthritis and Musculoskeletal Diseases (ESCEO) guideline, the Osteoarthritis Research Society International (OARSI) guideline, and American College of Rheumatology/Arthritis Foundation (ACR/AF) guideline in 2019, non-pharmacological treatments, including exercise, education, and weight loss (if overweight), should be initially prescribed [7–9]. Pharmacological agents such as non-steroidal anti-inflammatory drugs, intra-articular corticosteroids, and hyaluronic acids are subsequently added [7–9]. For patients with symptoms that can not be alleviated by conservative treatments, surgical intervention is further considered [10]. Among the various surgical interventions, total knee arthroplasty, unicompartmental knee arthroplasty, high tibial osteotomy (HTO), and distal femoral osteotomy are the most common types. For patients who want to preserve the knee joints and whose symptoms are caused by lower limb malalignment, HTO is a major trend [11–13].

Among the various HTO techniques, medial opening wedge HTO, lateral opening wedge HTO, medial closing wedge HTO, and lateral closing wedge HTO are the most common subtypes [13]. For medial compartment knee osteoarthritis with varus deformity, medial opening wedge HTO alleviates the symptoms by correcting the malalignment and redistributing the body weight loading [14, 15]. However, HTO alone does not aim to repair the structural damage of the knee. Therefore, orthobiologics, including platelet-rich plasma, bone marrow concentrate (BMC), and adipose-derived mesenchymal stem cells, have been applied for soft tissue regeneration and for their potential role in immunomodulation [16–18].

For medial opening wedge HTO, several studies have investigated the association of baseline demographic and radiographic features with clinical outcomes [19–23]. Although a few clinical studies and systematic reviews have investigated the efficacy of the combination of HTO and orthobiologics [24–26], few have reported and evaluated various structural, radiographic, and clinical factors that influenced the prognosis [27]. In our study, patients with medial compartment knee osteoarthritis were recruited. Medial opening wedge HTO was performed and BMC were postoperatively injected. Various demographic, structural, radiographic, and clinical factors that could potentially affect the outcomes were investigated to provide a more profound understanding of the efficacy of the combination therapy of medial opening wedge HTO and BMC.

## Materials and methods

### Patient enrollment

This prospective, open-label study was performed in line with the principles of the Declaration of Helsinki. The protocol was approved by the institutional review board (IRB) of Shin Kong Wu Ho-Su Memorial Hospital (Taipei, Taiwan) (IRB number: 20181006R). Prior to the recruitment of the study, the informed consent was obtained from all participants. We prospectively collected the data of 20 patients who underwent HTO in combination with BMC injection between June 2019 and May 2021. The inclusion criteria were as follows: (1) medial knee pain that could not be alleviated by non-pharmacological or pharmacological treatments; (2) radiographs showing moderate to severe medial compartment knee osteoarthritis with Kellgren-Lawrance (KL) grade II–IV; (3) patients with tibiofemoral angle less than valgus 5° or with varus deformity measured by standing anteroposterior radiographs; and (4) age between 20 and 70 years old. The exclusion criteria were as follows: (1) history of knee surgery; (2) history of stem cell transplantation in the knee; (3) other pathological diseases including rheumatoid arthritis, active knee infections, haemophilia, chronic anterior or posterior cruciate ligament instability; (4) participants with severe obesity (body mass index [BMI] ≥ 35); (5) active neuromuscular injury; (6) participants with poor health conditions that cannot tolerate surgical interventions; (7) participants with severe mental illness, developmental disability, inability to read consent forms, or unable to cooperate with the researchers; and (8) participants under pregnancy or breastfeeding. A total of 20 participants were recruited and included in the analysis. None of the participants were lost to follow-up.

### Surgical procedures

In the pre-operative phase, the desired correction angle and wedge size were measured and calculated from standing radiographs. Under endotracheal tube intubation general anaesthesia, the participant was placed in the supine position on a radiolucent operating table with a tourniquet applied. A medial longitudinal skin incision was made just distal to the joint line. After the pes anserinus was detached from the tibia, the superficial medial collateral ligament was exposed. The patellar tendon was protected by anterior retraction. Medial opening wedge osteotomy was carried out with a custom-made cutting jig. After temporary fixation of the cutting jig with multiple Kirschner-pins, the sawing was performed. The open wedge was subsequently filled with bone allograft, and a proximal medial tibia locking plate (APlus™) was used to fix the osteotomy.

### Preparation of the BMC

Prior to bone marrow collection, 1000–1500 IU/mL heparin was used to flush and rinse all the instruments. 1.5 ml acid-citrate-dextrose solution was then added to a 10 ml syringe as an anticoagulant. Subsequently, 8.5 ml bone marrow was slowly aspirated. A total of 30–40 ml bone marrow was extracted by iliac crest aspiration. The bone marrow aspirate was centrifuged using A-BMC (Aeon™) to autologous BMC. After the HTO operation, 4–6 ml bone marrow aspiration concentrate was injected into the knee joint.

### Outcome measurement

Radiographic outcomes of anatomical femorotibial angle and posterior tibial slope angle were measured at baseline and 12 months post-intervention. The measurements of anatomical femorotibial angle and posterior tibial slope angle were demonstrated in Supplementary Fig. 1. The clinical outcomes of pain (visual analogue scale [VAS]) and global function (Western Ontario and McMaster Universities Arthritis Index [WOMAC] and The Knee injury and Osteoarthritis Outcome Score [KOOS]) were obtained at baseline, 1 month, 3 months, 6 months, and 12 months postintervention.

### Statistical analysis

Shapiro–Wilk test was used to check the normality of the data. To evaluate the differences of the structural and radiological outcomes before and after HTO, paired Wilcoxon signed-rank tests and paired T tests were used for non-normally and normally distributed data, respectively. To evaluate the differences of the clinical outcomes at baseline and different follow-up time points, Friedman tests and repeated measures ANOVA (analyses of variance) were performed for non-normally and normally distributed data, respectively. For repeated measures ANOVA, Mauchly’s tests were conducted to check if the sphericity assumption was met, and Greenhouse–Geisser corrections were applied if the sphericity assumption was violated. Paired Wilcoxon signed-rank tests and paired T tests with Bonferroni multiple testing correction method were used as post hoc analyses for Friedman tests and repeated measures ANOVA, respectively. Data were reported as mean ± standard deviation (SD) or median (interquartile range [IQR]).

To model the change of the outcomes during the follow-up period, both linear and nonlinear mixed-effects models were used. The models with lower Akaike information criterion with correction for small sample sizes (AICc) and Bayesian information criterion (BIC) were considered to better fit the data. To further examine the factors that potentially influenced the treatment effects, covariates including age, sex, BMI, KL grade, femorotibial angle at baseline, posterior tibial slope angle at baseline, and symptoms severity at baseline were included in the models. Stepwise regressions, AICc, and BIC were used to select the optimal multivariate mixed-effects models. To validate the robustness of the multivariate models, the collinearity between the covariates were tested for significance. The values of correlation coefficient between 0 and 0.3 (0 and − 0.3) indicate a weak positive (negative) linear relationship, those between 0.3 and 0.7 (− 0.3 and − 0.7) indicate a moderate positive (negative) linear relationship, and those between 0.7 and 1.0 (− 0.7 and − 1.0) indicate a strong positive (negative) linear relationship [28]. The statistical analyses were all conducted in R (version 4.2.1), and the multivariate nonlinear mixed-effects models were performed using the “saemix” package.

## Results

### Baseline characteristics of the patients

Table 1 summarises the patient characteristics prior to the surgery. The patients comprised 12 men and 8 women with a mean age of 61.4 years old. The median Kellgren-Lawrence (KL) grade was 3. The patient had varus deformity with a mean anatomical femorotibial angle of 2.6° and a mean posterior tibial slope angle of 5.9°. The patients were generally overweight, with the mean BMI of 27.7 kg/m^2^. Regarding the symptoms at baseline, the median VAS was 62.5 mm, the median total WOMAC score was 40.2, and the mean total KOOS score was 210.9.

**Table 1:** Baseline characteristics of the patients

Variable	Value	Min–Max
Age, y	61.4 ± 7.1	43.3–67.9
Sex, male/female, n	12/8	-
Side of involvement, right/left, n	9/11	-
KL grade	Grade II: 3, Grade III: 11, Grade IV: 6	-
Femorotibial angle (°)	2.6 ± 4.6 (varus)	11.8 (varus)–2.7 (valgus)
Posterior tibial slope (°)	5.9 ± 3.5	0.8–13.8
BMI (kg/m^2^)	27.7 ± 6.0	21.1–45.8
VAS (mm)	62.5 (30.0)	5.0–95.0
WOMAC total	40.2 (21.8)	25.2–91.2
KOOS total	210.9 ± 65.5	59.4–346.3

Data presented as mean ± SD or median (IQR).The number of patients of each KL grade was presented

*Abbreviations*: *BMI* Body mass index, *IQR* Interquartile range, *KOOS* Knee injury and Osteoarthritis Outcome Score, *KL* Kellgren-Lawrence, *max* maximum; min, minimum, *SD* Standard deviation, *VAS* Visual analogue Scale, *WOMAC* Western Ontario and McMaster Universities Arthritis Index

### Radiographic outcomes

Both the anatomical femorotibial angle and posterior tibial slope angle showed significant improvement after medial opening wedge HTO (Table 2A). The varus deformities were corrected (*p* < 0.0001), with the mean femorotibial angle after surgery being 8.6° valgus. A slight increase of the mean posterior tibial slope from 5.9° to 7.6° was also observed (*p* = 0.04).

**Table 2:** Improvement of radiographic and clinical outcomes over the follow-up period

**A. Structural outcomes**						
**Time (month)**	**Femorotibial angle (°)**	**Posterior tibial slope (°)**				
0	-2.6 ± 4.6 (-11.8–2.7)	5.9 ± 3.5 (0.8–13.8)				
12	8.6 ± 2.3 (4.9–11.6)	7.6 ± 3.5 (1.5–14.9)				
Paired T test^*^	*p* < 0.0001	*p* = 0.04				
**B. VAS**						
**Time (month)**	**VAS (mm)**					
0	62.5 (30.0, 5.0–95.0)					
1	40.0 (20.0, 0.0–65.0)^a^					
3	25.0 (10.0, 0.0–40.0)^a,b^					
6	14.5 (10.0, 0.0–30.0)^a,b,c^					
12	10.0 (2.5, 0.0–20.0)^a,b,c^					
Friedman test	*χ*2(4) = 68.9, *p* < 0.0001					
**C. WOMAC**						
**Time (month)**	**Total**	**Pain**	**Stiffness**	**Physical function**		
0	40.2 (21.8, 25.2–91.2)	8.4 (5.6, 2.0–18.4)	5.4 (3.7, 0.8–7.6)	30.2 (14.8, 17.2–65.6)		
1	32.2 (18.0, 10.4–47.6)^a^	6.8 (3.8, 0.0–9.6)^a^	3.6 (2.0, 0.0–6.0)^a^	22.6 (10.6, 8.0–34.8)^a^		
3	22.8 (9.9, 4.4–40.8)^a,b^	4.8 (2.4, 0.0–7.2)^a,b^	2.4 (1.8, 0.8–4.0)^a,b^	15.4 (8.0, 1.6–29.6)^a,b^		
6	15.4 (9.4, 3.6–23.2)^a,b,c^	2.8 (1.8, 0.8–5.6)^a,b,c^	1.6 (0.6, 0.4–2.4)^a,b,c^	11.2 (6.4, 1.6–15.6)^a,b,c^		
12	10.0 (5.5, 2.8–20.4)^a,b,c,d^	2.0 (1.2, 0.0–4.4)^a,b,c,d^	0.8 (0.5, 0.4–1.6)^a,b,c^	6.8 (4.2, 1.2–14.4)^a,b,c^		
Friedman test	*χ*2(4) = 76.2, *p* < 0.0001	*χ*2(4) = 66.0, *p* < 0.0001	*χ*2(4) = 57.6, *p* < 0.0001	*χ*2(4) = 74.8, *p* < 0.0001		
**D. KOOS**						
**Time (month)**	**Total**	**Symptoms**	**Pain**	**Function, ADL**	**Function, sports and recreational activities**	**Quality of life**
0	210.9 ± 65.5 (59.3–346.3)	57.6 ± 13.3 (36.1–77.8)	52.6 ± 18.9 (5.6–86.1)	57.5 ± 15.3 (17.6–85.3)	31.3 ± 18.5 (0.0–70.0)	6.3 (18.8, 0.0–43.8)
1	257.9 ± 62.6 (174.0–364.4)	67.8 ± 12.3 (50.0–91.7)	61.4 ± 18.2 (33.3–91.7)	64.9 ± 12.0 (47.1–83.8)	38.5 ± 15.7 (20.0–70.0)	25.0 (10.9, 6.3–50.0)^a^
3	319.7 ± 58.4 (208.4–405.7)^a,b^	75.1 ± 8.0 (61.1–88.9)^a,b^	70.8 ± 13.7 (50.0–97.2)^a,b^	72.8 ± 14.3 (47.1–94.1)^a,b^	52.5 ± 14.2 (20.0–75.0)^a,b^	50.0 (9.4, 12.5–68.8)^a,b^
6	368.8 ± 43.8 (269.1–433.0)^a,b,c^	81.9 ± 7.0 (66.7–100.0)^a,b,c^	74.7 ± 11.0 (52.8–97.2)^a,b^	79.9 ± 12.1 (55.9–94.1)^a,b,c^	66.0 ± 8.5 (50.0–75.0)^a,b,c^	75.0 (20.3, 43.8–81.2)^a,b,c^
12	387.3 ± 36.6 (320.6–451.4)^a,b,c^	82.9 ± 6.0 (66.7–94.4)^a,b,c^	79.3 ± 11.2 (55.6–97.2)^a,b,c^	83.5 ± 8.1 (70.6–95.6)^a,b,c^	70.3 ± 9.5 (50.0–85.0)^a,b,c^	75.0 (6.3, 50.0–87.5)^a,b,c^
Repeated measures ANOVA	*F*(2.0, 38.3) = 75.2, *p* < 0.0001	*F*(2.1, 40.1) = 30.9, *p* < 0.0001	*F*(2.0, 38.2) = 26.3, *p* < 0.0001	*F*(1.7, 33.1) = 29.8, *p* < 0.0001	*F*(2.8, 52.5) = 45.9, *p* < 0.0001	*χ*2(4) = 72.0, *p* < 0.0001^†^

Data presented as mean ± SD (min–max) or median (IQR, min–max)

Femorotibial angle: varus = negative value, valgus = positive value

*Abbreviations*: *ADL* Activities of daily living, *ANOVA* Analysis of variance, *IQR* Interquartile range, *KOOS* Knee injury and Osteoarthritis Outcome Score, *max* maximum, *min* minimum, *SD* Standard deviation, *VAS* Visual analogue Scale, *WOMAC* Western Ontario and McMaster Universities Arthritis Index

^*^ Because of the nonparametric testing, only *p* values were provided

^†^ Friedman test used due to non-normal distribution of the data

^a^ Significant difference from baseline

^b^ Significant difference from 1 month post-intervention

^c^ Significant difference from 3 months post-intervention

^d^ Significant difference from 6 months post-intervention

### Improvement of clinical outcomes over the follow-up period

**Fig. 1:**
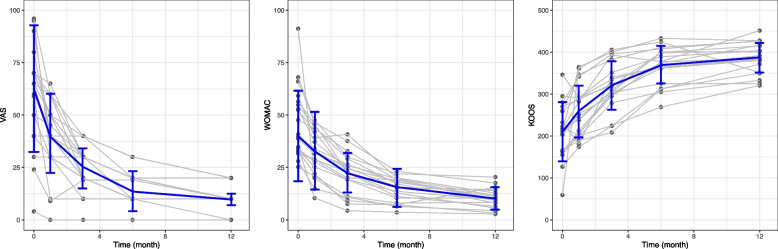
The trajectory of clinical symptom improvement over the follow-up duration. Each dot represents an outcome measure. The trajectories of symptom improvement of the patients are connected by grey lines. The blue lines represent the mean ± standard deviation or median ± interquartile range. Abbreviations: VAS, visual analogue scale; WOMAC, Western Ontario and McMaster Universities Arthritis Index; KOOS, The Knee injury and Osteoarthritis Outcome Score

Based on the results of the Friedman tests and repeated measures ANOVA, VAS, WOMAC, KOOS, and their respective subscales all showed significant improvement over the follow-up period (Table 2B–D and Fig. 1). Furthermore, the improvement of the symptoms increased with the progress of time. According to the post hoc tests, most of the outcomes improved significantly compared to those at the previous follow-up time points. However, VAS and KOOS revealed no significant differences between the scores at 6 months and those at 12 months after the surgery (Tables 2B and D). The results indicated a possible plateau in pain reduction and function improvement after 6 months of follow-up.

### Nonlinear mixed-effects model with asymptotic regression

Because a plateau was observed in symptom improvement as the follow-up duration increased, linear mixed-effects models may be unsuitable to fit the data. Nonlinear mixed-effects models with asymptotic regression were considered. The asymptotic regression can be described as the following equation:

$$\Delta (t)=a+({a}_{0}-a){e}^{-rt}$$

$t$ represents the duration of follow-up. $\Delta (t)$ represents the differences between patients’ post-intervention scores at follow-up time $t$ and baseline scores. ${a}_{0}$ represents the $\Delta$ at $t=0$ and should by definition be close to 0. $a$ represents the asymptote, which indicates the extent of symptom reduction when the plateau was reached. $r$ is the natural logarithm of the decline rate constant. To validate the superiority of the asymptotic regression, both the linear and nonlinear mixed-effects models were performed, and their AICc and BIC values were compared. We further considered other nonlinear models, including power regressions and polynomial regressions, for model fit. The nonlinear mixed-effects models with asymptotic regressions showed the best fit to the trajectory of the symptom improvement (Supplementary Table 1).

### Factors associated with greater symptom improvement

Based on the asymptotic regression, we further consider the effect of other covariates, including age, sex, BMI, KL grade, anatomical femorotibial angle at baseline, posterior tibial slope angle at baseline, and symptoms severity at baseline, on modifying the asymptote, thereby influencing the extent of symptom reduction and the rate of symptom improvement. The multivariate nonlinear mixed-effects model can be described as the following equation:

$$\Delta (t)=(a+\sum_{i}{\beta }_{i}{C}_{i})+\left[{a}_{0}-(a+\sum_{i}{\beta }_{i}{C}_{i})\right]{e}^{-rt}$$

**Fig. 2:**
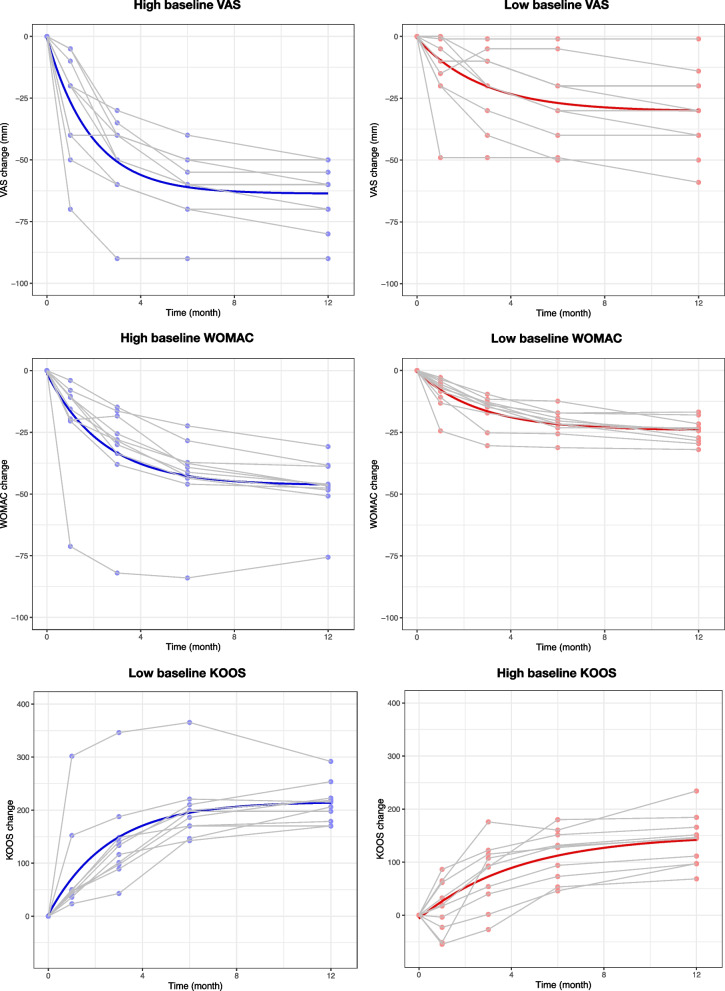
The regression lines of the patients with baseline symptom severity above and below the 50th percentiles. Each dot represents an outcome measure. The trajectories of symptom improvement of the patients are connected by grey lines. The blue and red lines represent the regression lines. Abbreviations: VAS, visual analogue scale; WOMAC, Western Ontario and McMaster Universities Arthritis Index; KOOS, The Knee injury and Osteoarthritis Outcome Score

$C$ represents the covariates. A linear relationship was assumed between the covariates and their effects on $a$, with $\beta$ implicating the correlation coefficient of the linearity. Using the method of stepwise regression to construct multivariate nonlinear mixed-effects models (Supplementary Table 2–4), symptom severity at baseline and KL grade were significantly correlated with all the three outcomes (Table 3A–C). To more clearly visualise the effects of baseline symptom severity on the treatment outcomes, the regression lines of the patients with baseline symptom severity above and below the 50th percentiles were respectively constructed (Fig. 2). Patients with more severe symptoms at baseline experienced more rapid and greater symptom relief. Lower KL grades were also positively correlated with symptom improvement (Table 3A–C). In addition to baseline symptoms and KL grades, the WOMAC and KOOS questionnaire reported better global function improvement in patients with lower BMI (Table 3B and C), and those with more prominent baseline varus deformity tended to benefit more in VAS and KOOS outcomes (Table 3A and C). Furthermore, sex and age may play a role in influencing the extent of pain reduction (Table 3A) and physical function advancement (Table 3C), respectively.

**Table 3:** Covariates in nonlinear mixed-effects models

**A. VAS**			
**Nonlinear mixed-model with covariates** $\Delta (t)=(a+\sum_{i}{\beta }_{i}{C}_{i})+\left[{a}_{0}-(a+\sum_{i}{\beta }_{i}{C}_{i})\right]{e}^{-rt}$			
**Parameter**	**Estimate**	**[95% CI]**	***P* value**
$a$	-9.32	[-20.75, 2.11]	-
${\beta }_{Sex}$	5.95	[0.99, 10.91]	0.009
${\beta }_{KL grade}$	4.69	[1.34, 8.04]	0.003
${\beta }_{FTA}$	0.45	[0.02, 0.88]	0.03
${\beta }_{PTS}$	-1.25	[-1.88, -0.63]	< 0.0001
${\beta }_{VAS baseline}$	-0.80	[-0.88, -0.72]	< 0.0001
${a}_{0}$	0.67	[-1.43, 1.70]	-
$r$	0.54	[0.36, 0.72]	-
AICc; BIC	693.07; 704.25		
**B. WOMAC**			
**Nonlinear mixed-model with covariates** $\Delta (t)=(a+\sum_{i}{\beta }_{i}{C}_{i})+\left[{a}_{0}-(a+\sum_{i}{\beta }_{i}{C}_{i})\right]{e}^{-rt}$			
**Parameter**	**Estimate**	**[95% CI]**	***P* value**
$a$	-14.53	[-26.68, -2.38]	-
${\beta }_{BMI}$	0.28	[0.01, 0.55]	0.04
${\beta }_{KL grade}$	3.27	[0.98, 5.56]	0.003
${\beta }_{WOMAC baseline}$	-0.87	[-0.88, -0.86]	< 0.0001
${a}_{0}$	0.13	[-0.81, 1.07]	-
$r$	0.47	[0.27, 0.67]	-
AICc; BIC	573.85; 583.44		
**C. KOOS**			
**Nonlinear mixed-model with covariates** $\Delta (t)=(a+\sum_{i}{\beta }_{i}{C}_{i})+\left[{a}_{0}-(a+\sum_{i}{\beta }_{i}{C}_{i})\right]{e}^{-rt}$			
**Parameter**	**Estimate**	**[95% CI]**	***P* value**
$a$	364.13	[211.94, 516.32]	-
${\beta }_{Age}$	1.58	[0.03, 3.13]	0.04
${\beta }_{BMI}$	-2.13	[-3.30, -0.06]	0.02
${\beta }_{KL grade}$	-10.06	[-20.08, -0.04]	0.04
${\beta }_{FTA}$	-5.51	[-7.85, -3.17]	< 0.0001
${\beta }_{KOOS baseline}$	-0.91	[-1.07, -0.75]	< 0.0001
${a}_{0}$	-8.18	[-16.80, 0.44]	-
$r$	0.40	[0.25, 0.55]	-
AICc; BIC	988.82; 1000.77		

Sex: male = 0, female = 1

Femorotibial angle: varus = negative value, vulgus = positive value

Abbreviations: ${\varvec{a}}$ asymptote, ${{\varvec{a}}}_{0}$ intercept, *AICc* Akaike information criterion with correction for small sample sizes, *BIC* Bayesian information criterion, *BMI* body mass index, ${{\varvec{C}}}_{{\varvec{i}}}$ the ith covariate, *CV* coefficient of variation, *FTA* femorotibial angle, *KL* Kellgren-Lawrence, *KOOS* Knee injury and Osteoarthritis Outcome Score, *PTS* posterior tibial slope, *r* natural logarithm of the rate constant, *SE* standard error, *t* follow-up time, *VAS* Visual analogue Scale, *WOMAC* Western Ontario and McMaster Universities Arthritis Index, ${{\varvec{\beta}}}_{{\varvec{i}}}$ correlation coefficient of the ith covariate, ∆ difference between patients’ postintervention and baseline scores

To validate the robustness of the multivariate models, the correlation coefficients among the covariates were calculated and tested for significance (Supplementary Fig. 1). Although most of the covariates showed no significant collinearity, moderate to strong collinearity was observed between sex and WOMAC at baseline and between sex and KOOS at baseline. However, sex was removed from the final model of WOMAC and KOOS using stepwise regression. The collinearity was therefore unlikely to influence the results. Moderate collinearity was also observed among some of the structural and radiographic factors. The effects of these factors should be cautiously interpreted.

### Adverse events

During the entire trial period, none of the patients reported infections, rejections, or other adverse events.

## Discussion

In our study, the combination of medial opening wedge HTO and BMC significantly reduced pain and improved global function in patients with medial compartment knee osteoarthritis and varus deformity. The improvement of clinical symptoms reached a plateau 6 months after the surgery. Using multivariate nonlinear mixed-effects models with asymptomatic regressions, symptom severity at baseline and KL grades were significantly correlated with the prognosis. BMI, femorotibial angle, age, and sex may also play a role in influencing the extent of symptom relief.

Medial opening wedge HTO is indicated for medial compartment knee osteoarthritis with varus deformity [29, 30]. By adjusting the femorotibial angle, the weight bearing of the knee can be redistributed [14, 15]. Although no consensus has been reached in terms of the optimal alignment in medial opening wedge HTO, 8–10° vulgus of the post-operative anatomical femorotibial angle was often suggested [31, 32]. In our study, the mean anatomical femorotibial angle was adjusted to 8.6° vulgus postoperatively, which indicated an appropriate correction of the malalignment. Moreover, our study indicated that patients with more severe pre-operative varus deformity experienced better post-operative outcomes, which further confirmed the benefits of medial opening wedge HTO on medial compartmental knee osteoarthritis. In addition to femorotibial angle, a slight increase in posterior tibial slope angle by 1.7° was observed. The result is in line with a previous meta-analysis, where the posterior tibial slope angle averagely increased 2.02° (95% confidence interval, 1.38° to 2.66°) after opening wedge HTO [33].

Prior to our study, some clinical studies have revealed the potential benefits of the combination of HTO and orthobiologics injection [24, 25, 27, 34–37]. However, few have investigated factors that could potentially affect the clinical outcomes [27]. In a study by Kim et al. [27], the effects of several factors on Lysholm score and KOOS after HTO and BMC injection were evaluated. Patients with higher KL grades and over 70 years old were significantly correlated with unsatisfactory clinical outcomes, whereas the association between other factors and clinical outcomes were not observed. In our study, the analyses of KL grades yielded similar results to those of the study by Kim et al.[27] However, contrary to their findings, older age may be related to better KOOS improvement, but due to the small sample size and the potential overfit of the stepwise regression in this study, the result could not be definitively confirmed. Moreover, patients > 70 years old were not included in this study. The results of the two studies may therefore not be comparable. In addition to KL grade and age, higher BMI was associated with lesser WOMAC and KOOS improvement. Although prior studies of HTO in combination with orthobiologics did not report similar results of BMI [27], the negative impact of overweight and obesity on the prognosis has been revealed in some of the previous HTO studies [21, 23, 38–40].

In our study, clinical symptom severity at baseline was included as a covariate in the nonlinear mixed-effects models, which was not investigated in the study by Kim et al.[27] According to previous cross-sectional studies, KL grades were not significantly correlated with symptoms of pain and physical function at baseline in patients with knee osteoarthritis, indicating absence of collinearity between radiographic and clinical factors [41, 42]. The tests for collinearity in our study also showed similar results. Therefore, the treatment strategies can be planned based on the baseline clinical features and functional status in addition to radiographic findings [41]. Based on the nonlinear mixed-effects models, patients with more severe pain or more limited physical function at baseline experienced more rapid and greater symptom relief. To the best of our knowledge, this is the first study of HTO in combination with BMC to indicate baseline symptom severity as a prognostic factor. In clinical practice, baseline clinical symptoms may also be considered in addition to structural and radiographic features prior to the intervention.

One of the highlights of our study is the use of multivariate nonlinear mixed-effects models. Using asymptotic regressions to model the trajectory of symptom improvement, the results showed some differences from those reported by the study by Kim et al. [27]. In the study by Kim et al.[27], the authors set a threshold and dichotomised the continuous scores into satisfactory and unsatisfactory outcomes. To model the dichotomous outcomes, multivariate logistic regressions were performed. However, in this study, we believe that the changes of symptom severity from baseline to post-intervention were of more clinical significance. Therefore, we did not dichotomise the scores and used asymptotic regressions to more precisely model the continuous change of the outcomes.

Our study has some limitations. First, there was no control group; thus, the comparison between HTO alone and the combination of HTO and BMC could not be performed. Furthermore, the analyses in this study may suffer from limitations of observational investigations without control groups, including bias arising from regression to the mean. Regression to the mean is a statistical phenomenon where the patients with relatively lower symptom severity at baseline tend to experience greater symptom severity nearer the true mean in the follow-up period, and vice versa, thereby making natural variation in repeated data look like real change [43]. However, because the patients in this study all experienced improvement in the outcomes, it may be unlikely that the effect of regression to the mean contributed to the overall effect estimates. Second, the sample size of this study was relatively small, which may not yield enough statistical power. The patients also had various disease severity and different KL grades. However, we did not perform subgroup analyses based on KL grades because this may further hinder the statistical power of this study. Third, to construct the multivariate nonlinear mixed-effects models, stepwise regressions were adopted, but the method is prone to overfitting. Therefore, we only focused on the factors that showed significant correlations with multiple outcomes. For those that showed significant correlation with only one outcome, the results should be cautiously interpreted. Fourth, because of the non-randomisation design of this study, selection bias may occur. However, because there were no missing data due to loss to follow-up, incomplete data collection, or exclusion from analysis, the probability of the existence of selection bias was low. Fifth, patients with more than 70 years of age were not included in this study; therefore, our result may not be able to be generalised to patients > 70 years old.

In conclusion, this is the first clinical study of medial opening wedge HTO in combination with BMC injection to demonstrate the importance of baseline symptom severity on the prognosis. In clinical practice, we suggest that the evaluation of clinical features and functional status of the patients be more emphasised. Studies with larger sample size and longer follow-up duration are also warranted to validate the current evidence.

## Supplementary Information

**Additional file 1:** **Supplementary Table 1.** Comparison of linear and nonlinear mixed-effects models. **Supplementary Table 2.** Stepwise regression in VAS. **Supplementary Table 3.** Stepwise regression in WOMAC. **Supplementary Table 4.** Stepwise regression in KOOS. **Supplementary Figure 1.** Demonstration of the measurement of anatomical femorotibial angle and posterior tibial slope angle. **Supplementary Figure 2.** Collinearity of the covariates in multivariate nonlinear mixed-effects models. 

## Data Availability

All data generated or analysed during this study are included in this published article and its supplementary information files.
