# Supplementary material for: Using multivariate nonlinear mixed-effects model to investigate factors influencing symptom improvement after high tibial osteotomy in combination with bone marrow concentrate injection for medial compartment knee osteoarthritis: a prospective, open-label study
Source: BMC Musculoskelet Disord. 2023 Mar 20;24:208. doi: 10.1186/s12891-023-06314-z (PMC10026441; doi:10.1186/s12891-023-06314-z)
Supplement: Supplementary file 1 — Additional file 1: Supplementary Table 1. Comparison of linear and nonlinear mixed-effects models. Supplementary Table 2. Stepwise regression in VAS. Supplementary Table 3. Stepwise regression in WOMAC. Supplementary Table 4. Stepwise regression in KOOS. Supplementary Figure 1. Demonstration of the measurement of anatomical femorotibial angle and posterior tibial slope angle. Supplementary Figure 2. Collinearity of the covariates in multivariate nonlinear mixed-effects models. [file 12891_2023_6314_MOESM1_ESM.docx]

**Supplementary Materials**

Supplementary Table 1. Comparison of linear and nonlinear mixed-effects models.

Supplementary Table 2. Stepwise regression in VAS.

Supplementary Table 3. Stepwise regression in WOMAC.

Supplementary Table 4. Stepwise regression in KOOS.

Supplementary Figure 1. Demonstration of the measurement of anatomical femorotibial angle and posterior tibial slope angle.

Supplementary Figure 2. Collinearity of the covariates in multivariate nonlinear mixed-

effects models.

**Supplementary Table 1. Comparison of linear and nonlinear mixed-effects models.**

| **A. VAS** |  |  |  |  |  |  |
| --- | --- | --- | --- | --- | --- | --- |
| **Linear mixed-effects model**  $\Delta\left( t \right)=\beta_{0}+\beta_{1}t$ | | |  | **Nonlinear mixed-effects model (asymptotic regression)**  $\Delta\left( t \right)=a+(a_{0}-a)e^{-rt}$ | | |
| **Parameter** | **Estimate** | **[95%CI]** |  | **Parameter** | **Estimate** | **[95%CI]** |
| $\beta_{0}$ | -14.09 | [-22.13, -6.05] |  | $a$ | -49.89 | [-59.16, -40.62] |
| $\beta_{1}$ | -3.39 | [-4.10, -2.68] |  | $a_{0}$ | 0.90 | [-1.22, 3.02] |
|  |  |  |  | $r$ | 0.56 | [0.36, 0.76] |
| AICc; BIC | 870.61; 880.91 | |  | AICc; BIC | 735.95; 743.26 | |
|  |  |  |  |  |  |  |
| **Nonlinear mixed-effects model (power regression)**  $\Delta\left( t \right)=\beta_{0}+\beta_{1}t^{0.5}$ | | |  | **Nonlinear mixed-effects model (power regression)**  $\Delta\left( t \right)=\beta_{0}+\beta_{1}t^{0.25}$ | | |
| **Parameter** | **Estimate** | **[95%CI]** |  | **Parameter** | **Estimate** | **[95%CI]** |
| $\beta_{0}$ | -4.70 | [-9.11, -0.29] |  | $\beta_{0}$ | 2.00 | [-1.14, 5.14] |
| $\beta_{1}$ | -14.10 | [-17.59, -10.61] |  | $\beta_{1}$ | -26.90 | [-33.47, -20.43] |
| AICc; BIC | 814.94; 815.91 | |  | AICc; BIC | 772.15, 777.02 | |
|  |  |  |  |  |  |  |
| **Nonlinear mixed-effects model (quadratic regression)**  $\Delta\left( t \right)=\beta_{0}+\beta_{1}t+\beta_{2}t^{2}$ | | |  | **Nonlinear mixed-effects model (cubic regression)**  $\Delta\left( t \right)=\beta_{0}+\beta_{1}t+\beta_{2}t^{2}+\beta_{3}t^{3}$ | | |
| **Parameter** | **Estimate** | **[95%CI]** |  | **Parameter** | **Estimate** | **[95%CI]** |
| $\beta_{0}$ | -4.09 | [-10.71, 2.53] |  | $\beta_{0}$ | -5.86 | [-12.84, 1.12] |
| $\beta_{1}$ | -11.14 | [-13.10, -9.18] |  | $\beta_{1}$ | -9.10 | [-14.43, -3.77] |
| $\beta_{2}$ | 0.63 | [0.48, 0.78] |  | $\beta_{2}$ | 0.28 | [-1.01, 1.57] |
|  |  |  |  | $\beta_{3}$ | 0.02 | [-0.02, 0.06] |
| AICc; BIC | 834.81, 841.51 | |  | AICc; BIC | 841.76, 850.31 | |
|  |  |  |  |  |  |  |
| **B. WOMAC** |  |  |  |  |  |  |
| **Linear mixed-effects model**  $\Delta\left( t \right)=\beta_{0}+\beta_{1}t$ | | |  | **Nonlinear mixed-effects model (asymptotic regression)**  $\Delta\left( t \right)=a+(a_{0}-a)e^{-rt}$ | | |
| **Parameter** | **Estimate** | **[95%CI]** |  | **Parameter** | **Estimate** | **[95%CI]** |
| $\beta_{0}$ | -9.23 | [-14.82, -3.64] |  | $a$ | -37.90 | [-44.64, -31.16] |
| $\beta_{1}$ | -2.60 | [-3.07, -2.36] |  | $a_{0}$ | -0.05 | [-0.99, 0.89] |
|  |  |  |  | $r$ | 0.47 | [0.27, 0.67] |
| AICc; BIC | 799.87; 804.85 | |  | AICc; BIC | 621.12; 627.78 | |
|  |  | |  |  |  | |
| **Nonlinear mixed-effects model (power regression)**  $\Delta\left( t \right)=\beta_{0}+\beta_{1}t^{0.5}$ | | |  | **Nonlinear mixed-effects model (power regression)**  $\Delta\left( t \right)=\beta_{0}+\beta_{1}t^{0.25}$ | | |
| **Parameter** | **Estimate** | **[95%CI]** |  | **Parameter** | **Estimate** | **[95%CI]** |
| $\beta_{0}$ | -2.80 | [-6.52, 0.92] |  | $\beta_{0}$ | 0.73 | [-3.07, 1.64] |
| $\beta_{1}$ | -10.50 | [-12.58, -8.42] |  | $\beta_{1}$ | -19.16 | [-23.53, -14.67] |
| AICc; BIC | 744.27; 749.25 | |  | AICc; BIC | 709.79, 714.77 | |
|  |  | |  |  |  | |
| **Nonlinear mixed-effects model (quadratic regression)**  $\Delta\left( t \right)=\beta_{0}+\beta_{1}t+\beta_{2}t^{2}$ | | |  | **Nonlinear mixed-effects model (cubic regression)**  $\Delta\left( t \right)=\beta_{0}+\beta_{1}t+\beta_{2}t^{2}+\beta_{3}t^{3}$ | | |
| **Parameter** | **Estimate** | **[95%CI]** |  | **Parameter** | **Estimate** | **[95%CI]** |
| $\beta_{0}$ | -3.56 | [-8.71, 1.59] |  | $\beta_{0}$ | -4.48 | [-10.11, 1.15] |
| $\beta_{1}$ | -7.41 | [-8.76, -6.06] |  | $\beta_{1}$ | -5.84 | [-9.74, -1.94] |
| $\beta_{2}$ | 0.43 | [0.32, 0.54] |  | $\beta_{2}$ | 0.04 | [-0.90, 0.98] |
|  |  |  |  | $\beta_{3}$ | 0.02 | [-0.03, 0.07] |
| AICc; BIC | 761.55; 768.52 | |  | AICc; BIC | 771.56; 780.52 | |
|  |  |  |  |  |  |  |
| **C. KOOS** |  |  |  |  |  |  |
| **Linear mixed-effects model**  $\Delta\left( t \right)=\beta_{0}+\beta_{1}t$ | | |  | **Nonlinear mixed-effects model (asymptotic regression)**  $\Delta\left( t \right)=a+(a_{0}-a)e^{-rt}$ | | |
| **Parameter** | **Estimate** | **[95%CI]** |  | **Parameter** | **Estimate** | **[95%CI]** |
| $\beta_{0}$ | 36.83 | [12.72, 60.94] |  | $a$ | 199.80 | [172.45, 227.15] |
| $\beta_{1}$ | 14.13 | [11.78, 16.48] |  | $a_{0}$ | -6.80 | [-14.95, 1.35] |
|  |  |  |  | $r$ | 0.40 | [0.24, 0.56] |
| AICc; BIC | 1109.68; 1114.66 | |  | AICc; BIC | 1010.79; 1017.76 | |
|  |  | |  |  |  | |
| **Nonlinear mixed-effects model (power regression)**  $\Delta\left( t \right)=\beta_{0}+\beta_{1}t^{0.5}$ | | |  | **Nonlinear mixed-effects model (power regression)**  $\Delta\left( t \right)=\beta_{0}+\beta_{1}t^{0.25}$ | | |
| **Parameter** | **Estimate** | **[95%CI]** |  | **Parameter** | **Estimate** | **[95%CI]** |
| $\beta_{0}$ | 1.80 | [-17.80, 21.40] |  | $\beta_{0}$ | -17.80 | [-33.28, -2.32] |
| $\beta_{1}$ | 55.60 | [46.58, 64.62] |  | $\beta_{1}$ | 100.30 | [-79.33, 121.27] |
| AICc; BIC | 1070.61; 1075.59 | |  | AICc; BIC | 1067.81; 1072.79 | |
|  |  | |  |  |  | |
| **Nonlinear mixed-effects model (quadratic regression)**  $\Delta\left( t \right)=\beta_{0}+\beta_{1}t+\beta_{2}t^{2}$ | | |  | **Nonlinear mixed-effects model (cubic regression)**  $\Delta\left( t \right)=\beta_{0}+\beta_{1}t+\beta_{2}t^{2}+\beta_{3}t^{3}$ | | |
| **Parameter** | **Estimate** | **[95%CI]** |  | **Parameter** | **Estimate** | **[95%CI]** |
| $\beta_{0}$ | 17.94 | [-7.21, 43.09] |  | $\beta_{0}$ | 15.23 | [-11.64, 42.10] |
| $\beta_{1}$ | 29.75 | [22.99, 36.51] |  | $\beta_{1}$ | 16.85 | [-2.28, 35.98] |
| $\beta_{2}$ | -1.37 | [-1.90, -0.84] |  | $\beta_{2}$ | 3.50 | [-1.15, 8.15] |
|  |  |  |  | $\beta_{3}$ | -0.31 | [-0.58, -0.04] |
| AICc; BIC | 1075.53; 1082.50 | |  | AICc; BIC | 1084.40; 1093.36 | |

Abbreviations: a, asymptote; $a_{0}$, intercept; AICc, Akaike information criterion with correction for small sample sizes; BIC, Bayesian information criterion; CI, confidence interval; r, natural logarithm of the rate constant; t, follow-up time; VAS, visual analogue Scale; WOMAC, Western Ontario and McMaster Universities Arthritis Index; KOOS, Knee injury and Osteoarthritis Outcome Score; $\beta_{0}$, intercept; $\beta_{1}$, slope; $\Delta$, change of the outcome from baseline.

**Supplementary Table 2. Stepwise regression in VAS.**

| **Step 0** |  |  |  |
| --- | --- | --- | --- |
| **Nonlinear mixed-effects model with covariates**  $\Delta\left( t \right)=(a+\sum_{i} \beta_{i}C_{i})+\left[ a_{0}-(a+\sum_{i} \beta_{i}C_{i}) \right]e^{-rt}$ | | | |
| **Parameter** | **Estimate** | **[95% CI]** | ***P* value** |
| $a$ | 3.82 | [-28.32, 35.96] | - |
| $\beta_{Age}$ | -0.14 | [-0.49, 0.21] | 0.22 |
| $\beta_{Sex}$ | 3.14 | [-2.58, 8.86] | 0.14 |
| $\beta_{BMI}$ | -0.01 | [-0.45, 0.43] | 0.48 |
| $\beta_{KL grade}$ | 5.15 | [-8.85, -1.45] | 0.003 |
| $\beta_{femorotibial angle}$ | 0.30 | [-0.19, 0.79] | 0.12 |
| $\beta_{posterior tibial slope}$ | -0.94 | [-1.60, -0.16] | 0.01 |
| $\beta_{VAS baseline}$ | -0.81 | [-0.93, -0.79] | <0.0001 |
| $a_{0}$ | 0.85 | [-1.27, 2.97] | - |
| $r$ | 0.55 | [0.35, 0.75] | - |
| AICc; BIC | 698.37; 709.93 | |  |
|  |  |  |  |
| **Step 1: remove age** |  |  |  |
| **Nonlinear mixed-model with covariates**  $\Delta\left( t \right)=(a+\sum_{i} \beta_{i}C_{i})+\left[ a_{0}-(a+\sum_{i} \beta_{i}C_{i}) \right]e^{-rt}$ | | | |
| **Parameter** | **Estimate** | **[95% CI]** | ***P* value** |
| $a$ | -11.99 | [-19.50, -4.48] | - |
| $\beta_{Sex}$ | 5.70 | [-10.66, -0.74] | 0.01 |
| $\beta_{BMI}$ | -0.02 | [-0.45, 0.41] | 0.46 |
| $\beta_{KL grade}$ | 5.50 | [2.13, 8.87] | <0.0001 |
| $\beta_{femorotibial angle}$ | 0.50 | [0.02, 0.98] | 0.02 |
| $\beta_{posterior tibial slope}$ | -1.09 | [-1.82, -0.36] | 0.01 |
| $\beta_{VAS baseline}$ | -0.80 | [-0.90, -0.70] | <0.0001 |
| $a_{0}$ | 0.87 | [1.23, 2.97] | - |
| $r$ | 0.55 | [0.37, 0.73] | - |
| AICc; BIC | 695.22; 707.00 | |  |
|  |  |  |  |
| **Step 2: remove BMI** |  |  |  |
| **Nonlinear mixed-model with covariates**  $\Delta\left( t \right)=(a+\sum_{i} \beta_{i}C_{i})+\left[ a_{0}-(a+\sum_{i} \beta_{i}C_{i}) \right]e^{-rt}$ | | | |
| **Parameter** | **Estimate** | **[95% CI]** | ***P* value** |
| $a$ | -9.32 | [-20.75, 2.11] | - |
| $\beta_{Sex}$ | 5.95 | [0.99, 10.91] | 0.009 |
| $\beta_{KL grade}$ | 4.69 | [1.34, 8.04] | 0.003 |
| $\beta_{femorotibial angle}$ | 0.45 | [0.02, 0.88] | 0.03 |
| $\beta_{posterior tibial slope}$ | -1.25 | [-1.88, -0.63] | <0.0001 |
| $\beta_{VAS baseline}$ | -0.80 | [-0.88, -0.72] | <0.0001 |
| $a_{0}$ | 0.67 | [-1.43, 1.70] | - |
| $r$ | 0.54 | [0.36, 0.72] | - |
| AICc; BIC | 693.07; 704.25 | |  |

Sex: male = 0, female = 1.

Femorotibial angle: varus (negative value), valgus (positive value).

Abbreviations: a, asymptote; $a_{0}$, intercept; AICc, Akaike information criterion with correction for small sample sizes; BIC, Bayesian information criterion; $C_{i}$, the ith covariate; CI, confidence interval; KL, Kellgren-Lawrence; r, natural logarithm of the rate constant; t, follow-up time; VAS, visual analogue Scale; $\beta_{i}$, slope of the ith covariate; $\Delta$, difference between patients’ postintervention and baseline scores.

**Supplementary Table 3. Stepwise regression in WOMAC.**

| **Step 0** |  |  |  |
| --- | --- | --- | --- |
| **Nonlinear mixed-model with covariates**  $\Delta\left( t \right)=(a+\sum_{i} \beta_{i}C_{i})+\left[ a_{0}-(a+\sum_{i} \beta_{i}C_{i}) \right]e^{-rt}$ | | | |
| **Parameter** | **Estimate** | **[95% CI]** | ***P* value** |
| $a$ | -2.11 | [-20.57, 16.35] | - |
| $\beta_{Age}$ | -0.15 | [-0.40, 0.10] | 0.13 |
| $\beta_{Sex}$ | 1.34 | [-3.72, 6.40] | 0.28 |
| $\beta_{BMI}$ | 0.30 | [0.03, 0.57] | 0.01 |
| $\beta_{KL grade}$ | 3.87 | [1.83, 5.91] | 0.0001 |
| $\beta_{femorotibial angle}$ | 0.43 | [0.11, 0.75] | 0.004 |
| $\beta_{posterior tibial slope}$ | 0.01 | [-0.38, 0.40] | 0.48 |
| $\beta_{WOMAC baseline}$ | -0.94 | [-1.06, -0.82] | <0.0001 |
| $a_{0}$ | 0.25 | [-0.79, 1.29] | - |
| $r$ | 0.48 | [0.28, 0.68] | - |
| AICc; BIC | 583.99; 592.95 | |  |
|  |  |  |  |
| **Step 1: remove sex** |  |  |  |
| **Nonlinear mixed-model with covariates**  $\Delta\left( t \right)=(a+\sum_{i} \beta_{i}C_{i})+\left[ a_{0}-(a+\sum_{i} \beta_{i}C_{i}) \right]e^{-rt}$ | | | |
| **Parameter** | **Estimate** | **[95% CI]** | ***P* value** |
| $a$ | -4.48 | [-25.63, 16.67] | - |
| $\beta_{Age}$ | -0.16 | [-0.40, 0.08] | 0.09 |
| $\beta_{BMI}$ | 0.28 | [-0.01, 0.57] | 0.05 |
| $\beta_{KL grade}$ | 3.54 | [1.27, 5.81] | 0.001 |
| $\beta_{femorotibial angle}$ | 0.37 | [0.04, 0.70] | 0.02 |
| $\beta_{posterior tibial slope}$ | 0.00 | [-0.47, 0.47] | 0.50 |
| $\beta_{WOMAC baseline}$ | -0.88 | [-0.98, -0.78] | <0.0001 |
| $a_{0}$ | 0.06 | [-0.90, 1.02] | - |
| $r$ | 0.47 | [0.27, 0.67] | - |
| AICc; BIC | 578.64; 590.63 | |  |
|  |  |  |  |
| **Step 2: remove posterior tibial slope** | |  |  |
| **Nonlinear mixed-model with covariates**  $\Delta\left( t \right)=(a+\sum_{i} \beta_{i}C_{i})+\left[ a_{0}-(a+\sum_{i} \beta_{i}C_{i}) \right]e^{-rt}$ | | | |
| **Parameter** | **Estimate** | **[95% CI]** | ***P* value** |
| $a$ | -2.08 | [-23.05, 18.89] | - |
| $\beta_{Age}$ | -0.19 | [-0.42, 0.04] | 0.08 |
| $\beta_{BMI}$ | 0.27 | [0.02, 0.52] | 0.03 |
| $\beta_{KL grade}$ | 3.50 | [-1.21, 5.79] | 0.001 |
| $\beta_{femorotibial angle}$ | 0.37 | [0.02, 0.72] | 0.03 |
| $\beta_{WOMAC baseline}$ | -0.88 | [-0.96, 0.80] | <0.0001 |
| $a_{0}$ | 0.29 | [-0.69, 1.27] | - |
| $r$ | 0.48 | [0.28, 0.68] | - |
| AICc; BIC | 576.82; 587.14 | |  |
|  |  | |  |
| **Step 3: remove age** | |  |  |
| **Nonlinear mixed-model with covariates**  $\Delta\left( t \right)=(a+\sum_{i} \beta_{i}C_{i})+\left[ a_{0}-(a+\sum_{i} \beta_{i}C_{i}) \right]e^{-rt}$ | | | |
| **Parameter** | **Estimate** | **[95% CI]** | ***P* value** |
| $a$ | -16.49 | [-28.49, -4.49] | - |
| $\beta_{BMI}$ | 0.29 | [0.02, 0.56] | 0.04 |
| $\beta_{KL grade}$ | 3.96 | [1.57, 6.35] | 0.001 |
| $\beta_{femorotibial angle}$ | 0.26 | [-0.13, 0.65] | 0.09 |
| $\beta_{WOMAC baseline}$ | -0.86 | [-0.87, -0.85] | <0.0001 |
| $a_{0}$ | 0.19 | [-0.77, 1.15] | - |
| $r$ | 0.47 | [0.27, 0.67] | - |
| AICc; BIC | 575.08; 585.60 | |  |
|  |  | |  |
| **Step 4: remove femorotibial angle** | |  |  |
| **Nonlinear mixed-model with covariates**  $\Delta\left( t \right)=(a+\sum_{i} \beta_{i}C_{i})+\left[ a_{0}-(a+\sum_{i} \beta_{i}C_{i}) \right]e^{-rt}$ | | | |
| **Parameter** | **Estimate** | **[95% CI]** | ***P* value** |
| $a$ | -14.53 | [-26.68, -2.38] | - |
| $\beta_{BMI}$ | 0.28 | [0.01, 0.55] | 0.04 |
| $\beta_{KL grade}$ | 3.27 | [0.98, 5.56] | 0.003 |
| $\beta_{WOMAC baseline}$ | -0.87 | [-0.88, -0.86] | <0.0001 |
| $a_{0}$ | 0.13 | [-0.81, 1.07] | - |
| $r$ | 0.47 | [0.27, 0.67] | - |
| AICc; BIC | 573.85; 583.44 | |  |

Sex: male = 0, female = 1.

Femorotibial angle: varus (negative value), valgus (positive value).

Abbreviations: a, asymptote; $a_{0}$, intercept; AICc, Akaike information criterion with correction for small sample sizes; BIC, Bayesian information criterion; $C_{i}$, the ith covariate; CI, confidence interval; KL, Kellgren-Lawrence; r, natural logarithm of the rate constant; t, follow-up time; WOMAC, Western Ontario and McMaster Universities Arthritis Index; $\beta_{i}$, slope of the ith covariate; $\Delta$, difference between patients’ postintervention and baseline scores.

**Supplementary Table 4. Stepwise regression in KOOS.**

| **Step 0** |  |  |  |
| --- | --- | --- | --- |
| **Nonlinear mixed-model with covariates**  $\Delta\left( t \right)=(a+\sum_{i} \beta_{i}C_{i})+\left[ a_{0}-(a+\sum_{i} \beta_{i}C_{i}) \right]e^{-rt}$ | | | |
| **Parameter** | **Estimate** | **[95% CI]** | ***P* value** |
| $a$ | 313.53 | [123.70, 503.36] | - |
| $\beta_{Age}$ | 1.47 | [-0.51, 3.45] | 0.08 |
| $\beta_{Sex}$ | 20.26 | [-13.04, 53.56] | 0.12 |
| $\beta_{BMI}$ | -1.59 | [-3.91, 0.73] | 0.09 |
| $\beta_{KL grade}$ | -10.19 | [-25.95, 5.57] | 0.10 |
| $\beta_{femorotibial angle}$ | -5.57 | [-8.47, -2.67] | <0.0001 |
| $\beta_{posterior tibial slope}$ | -0.10 | [-2.95, 2.75] | 0.47 |
| $\beta_{KOOS baseline}$ | -0.74 | [-0.98, -0.50] | <0.0001 |
| $a_{0}$ | -8.10 | [-16.45, 0.25] | - |
| $r$ | 0.42 | [0.25, 0.59] | - |
| AICc; BIC | 991.52; 1005.46 | |  |
|  |  |  |  |
| **Step 1: remove sex** |  |  |  |
| **Nonlinear mixed-model with covariates**  $\Delta\left( t \right)=(a+\sum_{i} \beta_{i}C_{i})+\left[ a_{0}-(a+\sum_{i} \beta_{i}C_{i}) \right]e^{-rt}$ | | | |
| **Parameter** | **Estimate** | **[95% CI]** | ***P* value** |
| $a$ | 374.52 | [214.09, 534.95] | - |
| $\beta_{Age}$ | 1.30 | [-0.57, 3.17] | 0.09 |
| $\beta_{BMI}$ | -2.23 | [-0.13, 4.33] | 0.02 |
| $\beta_{KL grade}$ | -12.58 | [-28.16, 3.00] | 0.06 |
| $\beta_{femorotibial angle}$ | -5.44 | [-8.30, -2.58] | 0.0001 |
| $\beta_{posterior tibial slope}$ | 0.52 | [-2.36, 3.40] | 0.36 |
| $\beta_{KOOS baseline}$ | -0.86 | [-1.04, -0.68] | <0.0001 |
| $a_{0}$ | -7.56 | [-16.18, 1.06] | - |
| $r$ | 0.41 | [0.25, 0.57] | - |
| AICc; BIC | 990.78; 1003.72 | |  |
|  |  |  |  |
| **Step 2: remove posterior tibial slope** | |  |  |
| **Nonlinear mixed-model with covariates**  $\Delta\left( t \right)=(a+\sum_{i} \beta_{i}C_{i})+\left[ a_{0}-(a+\sum_{i} \beta_{i}C_{i}) \right]e^{-rt}$ | | | |
| **Parameter** | **Estimate** | **[95% CI]** | ***P* value** |
| $a$ | 364.13 | [211.94, 516.32] | - |
| $\beta_{Age}$ | 1.58 | [0.03, 3.13] | 0.04 |
| $\beta_{BMI}$ | -2.13 | [-3.30, -0.06] | 0.02 |
| $\beta_{KL grade}$ | -10.06 | [-20.08, -0.04] | 0.04 |
| $\beta_{femorotibial angle}$ | -5.51 | [-7.85, -3.17] | <0.0001 |
| $\beta_{KOOS baseline}$ | -0.91 | [-1.07, -0.75] | <0.0001 |
| $a_{0}$ | -8.18 | [-16.80, 0.44] | - |
| $r$ | 0.40 | [0.25, 0.55] | - |
| AICc; BIC | 988.82; 1000.77 | |  |

Sex: male = 0, female = 1.

Femorotibial angle: varus (negative value), valgus (positive value).

Abbreviations: a, asymptote; $a_{0}$, intercept; AICc, Akaike information criterion with correction for small sample sizes; BIC, Bayesian information criterion; $C_{i}$, the ith covariate; CI, confidence interval; KL, Kellgren-Lawrence; KOOS, Knee injury and Osteoarthritis Outcome Score; r, natural logarithm of the rate constant; t, follow-up time; $\beta_{i}$, slope of the ith covariate; $\Delta$, difference between patients’ postintervention and baseline scores.

**Supplementary Figure 1. Demonstration of the measurement of anatomical femorotibial angle and posterior tibial slope angle.**

**A**

**B**

The anatomic axes of the femur and tibia are obtained from a line centred in the diaphysis of the bone. (A) The anatomical femorotiabial angle is the angle between the two axes. (B) The posterior tibial slope is the angle between the vertical line of the tibial anatomical axis and the tibial plateau tangent. Numbers in the figure: (1) femoral anatomical axis; (2) tibial anatomical axis; (3) tibial anatomical axis; (4) vertical line of the tibial anatomical axis; (5) the tangent line of the tibial plateau.

**Supplementary Figure 2. Collinearity of the covariates in multivariate nonlinear mixed-**

**effects models.**

The dots in the left lower part represent the original data of the patients. The graphs along the diagonal line indicate the distribution of the patient data. The numbers in the right upper part represent the correlation coefficients between each pair of the covariates.

*p <0.05, **p <0.01, ***p <0.001.

Sex: male = 0, female = 1.

Femorotibial angle: varus (negative value), valgus (positive value).

Abbreviations: BMI, body mass index; Corr, correlation coefficient; FTA, femorotibial angle; KL, Kellgren-Lawrence grade; KOOS_BL, Knee injury and Osteoarthritis Outcome Score at baseline; PTS, posterior tibial slope angle; VAS_BL, visual analogue scale at baseline; WOMAC_BL, Western Ontario and McMaster Universities Arthritis Index at baseline.
